# The role of BCL11B in hematological malignancy

**DOI:** 10.1186/2162-3619-1-22

**Published:** 2012-08-22

**Authors:** Xin Huang, Xin Du, Yangqiu Li

**Affiliations:** 1 Department of Hematology, Guangdong General Hospital (Guangdong Academy of Medical Sciences), Guangzhou, 510080, China; 2 Institute of Hematology, Medical College, Jinan University, Guangzhou, 510632, China; 3 Key Laboratory for Regenerative Medicine of Ministry of Education, Jinan University, Guangzhou, 510632, China

**Keywords:** BCL11B, Altered expression, Hematological malignancy, Apoptosis, Targeted therapy

## Abstract

The B-cell leukemia/lymphoma 11B (BCL11B) gene is a member of the BCL family which plays a crucial role in the development, proliferation, differentiation and subsequent survival of T cells. BCL11B gene alterations are related to malignant T cell transformation that occurs in hematological malignancies. Remarkably, the BCL11B gene is responsible for the regulation of the apoptotic process and cell proliferation. This review summarizes current data and knowledge concerning the alteration of BCL11B in hematological malignancies and its role as a potential target for therapies directed against T cell malignancies.

## Introduction

### The structure of BCL11B

#### The BCL11B gene

**Figure 1:**
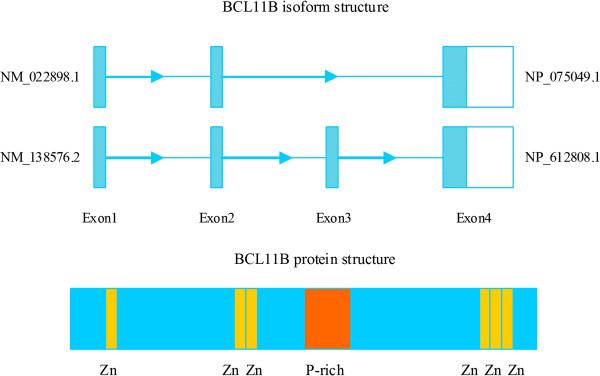
Schematic model of BCL11B gene isoforms structure (2 splice variants) and protein structure (the location of six zinc fingers).

B-cell leukemia/lymphoma 11B (BCL11B) was first described by Ed Satterwhite in 2001. The BCL11B gene is located on mouse chromosome 12 (52.0 cM) and human chromosome 14 (q32.1). Murine BCL11B shows 88% identity to the human BCL11B at nucleotide level. It has been successfully demonstrated that BCL11B expression begins in the early double negative 1 (DN 1) cell stage in the thymus, and is primarily expressed in T cells, thymocytes and brain tissue [1]. This gene was originally referred to as RIT1 (radiation-induced tumor suppressor gene 1) because BCL11B was isolated by scanning γ-ray-induced mouse thymic lymphomas for the loss of specific chromosomal DNA [2]. BCL11B is also known as CTIP2 (COUP-TF-interacting protein 2) because it was isolated for its interaction with the orphan nuclear receptor chicken ovalbumin upstream promoter transcription factor (COUP-TF) [3]. The BCL11B gene consists of 4 exons, and two alternatively spliced transcript variants, which encode distinct isoforms possessing or lacking exon 3, have been reported (Figure 1) [4,5]. 

### The BCL11B protein

BCL11B belongs to the Kruppel-like C_2_H_2_ type zinc finger transcription factor family that contains 6 C_2_H_2_ zinc fingers and proline-rich and acidic regions with 95% identity in their zinc finger domains [4]. BCL11B encodes two different isoforms consisting of 823 and 894 aa in humans (Figure 1). These structures include DNA binding and protein interacting regions. The long exon 4 comprises the 6 zinc-finger domains, and the 2^nd^ and 3^rd^ domains are responsible for DNA binding. Apart from the DNA binding region, BCL11B possesses domains responsible for interaction with proteins and protein complexes [5].

### BCL11B biological functions

The specific functions of this gene have yet to be determined. The number of BCL11B functional studies have recently been on the rise.

### Transcriptional regulator

As a transcription factor, BCL11B may be a bi-functional transcriptional regulator that acts as a repressor and transactivator [6]. BCL11B interacts with COUP-TF [3] and nucleosome remodeling and histone deacetylation complex (NuRD) [7], rendering it a potent transcriptional repressor. Genes encoding the cyclin-dependent kinase inhibitors p21/Cip2/Waf1 and p57/Kip2 are transcriptionally suppressed by BCL11B [8,9]. BCL11B directly interacts with the P2 promoter region of HDM2, which is the human homologue of MDM2 (mouse double minute 2) and inhibits the HDM2 promoter activity in a p53-dependent manner [10]. Conversely, interaction between BCL11B and the p300 coactivator at upstream site 1 (US1) in the IL-2 promoter results in transcriptional activation of IL-2 expression in activated T cells [11]. BCL11B enhances TCR/CD28-triggered NF-κB activation by up-regulating Cot kinase gene expression in T-cells [12].

### T-cell development and the maintenance of T-cell identity

BCL11B plays a key role in both T-cell development and subsequent maintenance of T-cell identity [13]. BCL11B is necessary for T lineage commitment in mice, and is specifically required in order to successfully repress natural killer cell-associated genes while down-regulating a battery of stem or progenitor cell genes at their pivotal commitment stage [6].

BCL11B-deficient mice demonstrate a block at their CD4-CD8- DN thymocytes development stage without impairment in any of the cells in the B- and γδ T-cell lineages. However, Bcl11b−/− thymocytes demonstrated unsuccessful Vβ to Dβ recombination, and lacked necessary pre-T cell receptor (TCR) complex expression on the cell surface; this was due to the absence of TCRβ mRNA expression. Further, the researchers observed massive apoptosis in the thymi of neonatal BCL11B−/− mice, results which indicate that BCL11B is a key regulator of the differentiation and survival of αβ T cells during thymocyte development [14]. Removal of the BCL11B transcription factor in double positive (DP) thymocytes leads to an early block in invariant natural killer T-cell (iNKT) development. Remarkably, these studies demonstrated a unique, previously undescribed role played by BCL11B in DP thymocytes, in addition to its already critical function in the positive selection of conventional CD4 and CD8 single-positive thymocytes [15]. T cells and mature T cells both acquire NK cell properties, transforming them into natural killer (NK) cells upon BCL11B deletion during culture. These reprogrammed cells possess unique properties of proliferation, cytokine dependency and target cell killing [1,16]. Our data revealed that after BCL11B up-regulation, naïve T cells increased their proliferation ability; predominantly in the Th differentiated subset [17], which may result from increased CXCL10 and CXCL11 expression. After BCL11B down-regulation, proliferation capacity substantially decreased, which could be related to changes in expression of the mitochondrial pathway genes CFLAR, CASP8, and CASP10. Moreover, we found that BCL11B was indispensable for Treg suppressor function, the maintenance of optimal Foxp3 and IL-10 gene expression, and the induction of Foxp3 expression in conventional CD4+ T cells in response to TGF-β and iTreg cell generation [18].

### Tumor suppressor

BCL11B has recently been identified as a tumor suppressor gene [5,19]. It has been demonstrated that BCL11B is a haplo-insufficient tumor suppressor which collaborates with all major T-cell, acute lymphoblastic leukemia (T-ALL) oncogenic lesions during human thymocyte transformation [19], the loss of a BCL11B allele provides susceptibility to mouse thymic lymphoma [20] and human T-ALL [21-23]. The absence of a BCL11B tumor suppressor resulted in vulnerability to DNA replication stress and damage [24]. Down-regulation of the BCL11B gene by small interfering RNA (siRNA) led to growth inhibition and apoptosis in a human T-ALL cell line, although not in normal mature T and CD34+ cells [25-27].

### BCL11B alterations in hematological malignancies

#### Deletion/mutation/disruption

The findings indicated that BCL11B haplo-insufficiency occurred across each of the major T-ALL molecular subtypes, including the early T-cell precursor, HOXA-positive, LEF1-inactivated, and TAL1-positive subtypes [19]. Loss-of-function BCL11B mutations contribute to mouse lymphomagenesis and thus potentially also to human cancer development [20]. Monoallelic BCL11B deletions or missense mutations were found in 9% (10/117) of a collection of T-ALL diagnostic specimens. There are at least six examples of intra-chromosomal interstitial BCL11B deletions caused by aberrant V (D) J recombination between non-antigen receptor loci, and five of these six are associated with lymphoid malignancy. Although the efficiency of these illegitimate V (D) J recombination events is less, by several orders of magnitude than at bonafide antigen receptor loci, such deletions result in devastating consequences, and represent a major cause of lymphoid malignancy [28]. Analysis of the D-J rearrangement at the TCRβ locus and cell surface markers after the γ-irradiation of BCL11B (KO/+) mice revealed that BCL11B allelic loss had occurred at the stage following V(D)J recombination. BCL11B heterozygosity can affect lymphomagenesis at the CD117^high^ DN2 stage which occurs after the CD117^high^ DN1 stage. In BCL11B (KO/+) thymocytes, the expression of recognized oncogenic protein β-catenin was increased. Subsequently, the BCL11B (KO/+) genotype contributes to clonal expansion and differentiation arrest, which is due in part to an increase in the β-catenin level [5,29]. Structural homology modeling revealed that several of the BCL11B mutations disrupted the structure of zinc finger domains which are necessary in order for this transcription factor to bind DNA.

### Rearrangement/juxtaposition

Cytogenetic studies in a patient with acute myeloid leukemia (AML) revealed that the sole karyotypic alteration of a half-cryptic rearrangement, which was identified as t (6; 14) (q25-q26; q32), involved the BCL11B gene [30]. A cryptic chromosome rearrangement, t (5;14)(q35.1;q32.2) was recently identified in a pediatric patient with acute lymphoblastic leukemia (ALL) [31-34], T/myeloid acute bilineal leukemia [35], and a pediatric T-ALL cell line (HPB-ALL); the rearrangement of which targets activation in HOX11L2/TLX3 at 5q35.1 through juxtaposition with a downstream region of BCL11B at 14q32.2 [36]. A rearrangement of NKX2-5-BCL11B was also found in T-cell lines PEER and CCRF-CEM, which resulted in a high level of NKX2-5 expression. NKX2-5 is a homeodomain-containing transcription factor expressed in the nucleus during embryonic development, which has been associated with T-ALL [37].

### Overexpression

In the human body, BCL11B over-expression is primarily found in T-ALL [25,26]. A comparison of genome profiles of acute and lymphoma types revealed BCL11B over-expression in the acute form, regardless of the 14q32 gain/amplification, but either low or no levels of this gene’s expression in the lymphomas; these results suggest that acute and lymphoma types are genomically distinct subtypes, which thus may develop tumors via distinct genetic pathways [38]. Apoptosis resistance triggered by BCL11B over-expression was found to be accompanied by chemo-resistance caused by the accumulation of T-ALL cells in the G1 phase [39].

### Methylation status

Based on a genome-wide analysis of aberrant DNA methylation in chronic lymphocytic leukemia (CLL) using methylated CpG island amplification (MCA), coupled with a promoter microarray, methylation status was confirmed through pyro-sequencing for 22 of these genes in 78 CLL patients, including BCL11B [40]. However, the contribution of inactivation of BCL11B in B-CLL cells requires further investigation.

### Targeted therapies

**Figure 2:**
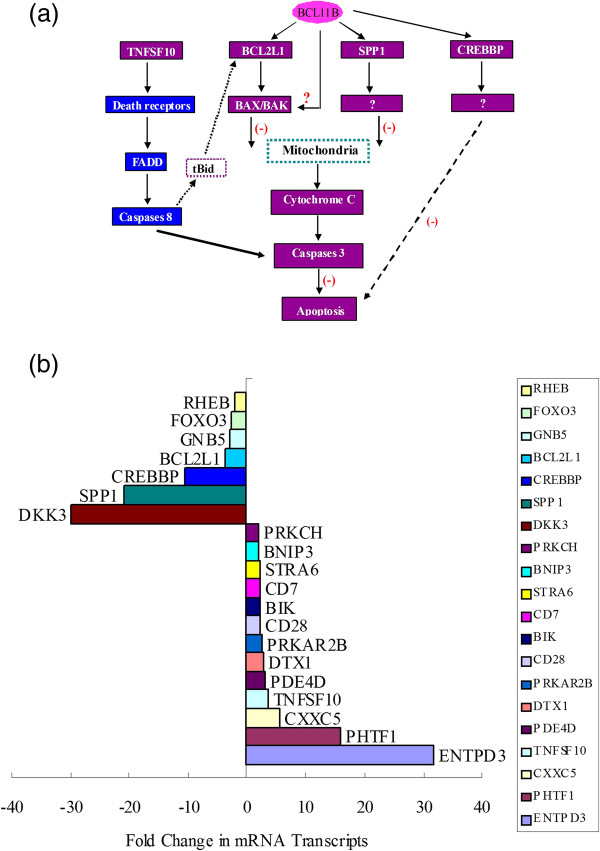
Schematic model of molecular mechanism of BCL11B pathways (A) and differentially expressed genes in BCL11B siRNA-treated T-ALL cells (B).

Based upon funding proposal data, BCL11B targeting may be considered a new therapeutic strategy for T-cell malignancies. BCL11B silencing by RNA interference in T-ALL significantly inhibited cell proliferation and induced apoptosis. The molecular mechanism behind this result may be related to several pathways which mainly include the apoptosis pathway (e.g., TNFSF10, BIK, BNIP3, and BCL2L1 genes) and the TGF beta pathway (e.g., SPP1 and CREBBP genes) (Figure 2A) [41,42]. Global gene expression profile analysis revealed that the PHTF1 (putative homeodomain transcription factor 1) gene increased 16-fold in BCL11B siRNA-treated T-ALL cells (Figure 2B). Further, proteome analysis verified that in fact new mechanisms of BCL11B-loss driven apoptosis may be related to the mitochondrial apoptotic pathway, and observed cleavage and fragments of known caspase targets may be involved: such as myosin, spectrin, vimentin, and ERM proteins, which were up-regulated and phosphorylated upon BCL11B down-regulation. Moreover, the levels of several proteins implicated in cell cycle entry, including DUT-N, CDK6, MCM4, MCM6, and MAT1, were elevated [43]. 

## Conclusions and future directions

To improve T-cell malignancy treatment strategies, further work is necessary in order to most accurately identify therapeutic targets. Gastrointestinal stromal tumor (GIST) research and clinical care sets are examples of such translational research, capable of transforming laboratory discoveries into successful clinical applications [44]. A promising therapeutic target for T-cell malignancy therapy can indeed be the result of this fundamental, mechanistic understanding of the role of BCL11B; its regulation of the apoptotic process, and its over-expression in leukemic T cells, using a monoclonal antibody, small molecule inhibitor (or siRNA). It should be noted, however, that the inhibitory effects may be limited for single agents, and its combination with chemotherapy may have a distinctly synergistic effect in killing the leukemic cells.

## Competing interests

The authors declare that they have no competing interests.

## Authors’ contributions

YQL directed and conceived the study and discussion. XH, XD and YQL were involved in manuscript preparation. All authors reviewed and assisted in revising the manuscript. All authors read and approved the final manuscript.
